# ‘Knowing how the machine works’: a novel framework for engaging with the political determinants of health

**DOI:** 10.1136/bmjgh-2025-019640

**Published:** 2025-08-21

**Authors:** Fritz Brugger, Joschka J Proksik, Olga Cambaço, Isaac Lyatuu, Philip Adongo, Martin Amogre Ayanore, Mirko S Winkler

**Affiliations:** 1 D-GESS, ETH Zürich, Zürich, Switzerland; 2 Social Science, Centro de Investigacao em Saude de Manhica, Maputo, Mozambique; 3 Ifakara Health Institute, Dar es Salaam, Tanzania, United Republic of; 4 School of Public Health, University of Ghana, Accra, Ghana; 5 Department of Health Policy Planning and Management, UHAS, Ho, Ghana; 6 Swiss Tropical and Public Health Institute, Allschwil, Switzerland; 7 University of Basel, Basel, Switzerland

**Keywords:** Public Health, Global Health, Health policy, Interdisciplinary Research

## Abstract

It is well established that *social* determinants influence people’s health and well-being. Yet, limited attention is given to the fact that social determinants are, in turn, the result of *political* determinants. The political struggle between different ideas, interests and power over public policies, that is, how societies shall function, results in formal and informal structures that work as a ‘sorting machine’: they determine how societies (re)distribute material resources and opportunities among their members, creating societal classes that face disparate health outcomes. Their visible and measurable characteristics are then labelled ‘social determinants’. Improving public health, therefore, needs engaging with the political determinants.

We contribute to this broader vision with a process-oriented, interdisciplinary framework. It first operationalises the analysis of political determinants of health to understand how, in a specific public health concern, politics shapes policy processes and causes differential health outcomes. Building on these insights, the framework then supports strategic engagement with the politics of policy processes, using agile methods to identify and promote more equitable and politically feasible policy options.

We illustrate our approach by reporting on an interdisciplinary 6-year research project in Burkina Faso, Ghana, Mozambique and Tanzania, examining how structural determinants contribute to unequal public health outcomes in industrial mining areas. Trade-offs between attracting mining firms and safeguarding public health have led to a patchwork of national legislation, global governance regimes and voluntary commitments, inviting an eclectic consideration of public health industrial mining. For instance, among over 560 mining projects across Africa, more than 90% of impact assessments were inaccessible. In the few that were, public health is only marginally considered, disproportionately harming vulnerable populations.

To strengthen the ‘structural competence’ and policy relevance of the public health discipline, our results suggest an institutionalised cooperation with political science in curricula, training and applied research.

**WHAT IS ALREADY KNOWN ON THIS TOPIC**

- While public health research recognises the role of social determinants and equity issues in shaping health outcomes, it frequently overlooks the political determinants, that is, how policies and political processes influence equity and social determinants.

**WHAT THIS STUDY ADDS**

- This study introduces a process-oriented framework that integrates political science and epidemiology to analyse political determinants of health and to engage with the politics of policy processes.

**HOW THIS STUDY MIGHT AFFECT RESEARCH, PRACTICE OR POLICY**

- The operationalisation of the framework is illustrated through its application to public health in industrial mining in four African countries.
- Building on the interdisciplinary collaboration between political science and public health scholars, the framework strengthens political analysis, advances policy engagement and expands methodological tools for public health research.
- This approach can help bridge the gap between evidence-based health interventions and the complex realities of policymaking, ultimately strengthening health equity efforts.

## Introduction

 It has become commonplace among public health experts to emphasise that social determinants influence the health and well-being of individuals and populations. However, it is less clear how social determinants of health are waned and waxed. Specifically, what determines attainable income, education and employment opportunities, access to health services, water, sanitation, the risk of food insecurity, the quality of housing, the experience of social exclusion, discrimination or the quality of the environment? It is much less common to assert that social determinants of health are the result of political contestation and (power)struggles over social, economic and environmental policies and the (re)distribution of material resources and opportunities across societal groups and classes.^1^

Limited attention has been given to the political context and policy contestation in a substantial portion of the literature on health determinants and public health analysis.^2 3^ Of 6200 policy studies into social determinants of health and health equity, only 7 were found to engage at least somewhat systematically with the underlying public policy mechanisms and processes.^4^ Evaluations of health system interventions are typically descriptive, rarely explanatory and often lack a political economy analysis^5^; the same holds for research into ‘health in all policies’.^6^

Prima facie, public health researchers may be reluctant to engage more deeply with politics because this is not their primary discipline. Public health specialists are trained in medicine, biology and other natural sciences, supplemented by sociological perspectives to enhance understanding of health equity. Arguably, they would need a much better understanding of politics and how to engage with politicians.^7^ Yet, the reasons go deeper. Public health research is grounded in a positivist epistemology and ontology, where measurable evidence is valued, and decision-making is informed by a linear, rationalist logic to which apolitical rational choice models or behavioural studies better conform.^8 9^ Political processes, in contrast, are non-linear, complex and often wicked, readily shaped by the discursive battle of ideas, interests and power.^10^ By its very nature, politics, including politics of health, has normative dimensions; it deliberates over the respective roles and responsibilities of the state, markets and individuals in solving public issues.^7 11^ Such fundamental differences translate into politics having ‘a very bad name’^12^ and being perceived ‘as a subway’s third rail: avoid touching it, lest you get burned’.^10^ Unsurprisingly, most public health experts concerned about policies tend to resort to a narrow technocratic approach, ‘setting out why an evidence-based action should be done without asking how it might be done’.^12^

In contrast to mainstream public health scholarship, critical public health research^13^,^15^ unpacks how interests, influence and the power of framing shape the policies adopted and the outcomes produced.^16^,^18^ This literature examines the effects of neo-liberal reforms,^14^ global governance of health,^19^ trade and investment agreements,^20^ financialisation^21^ and digitalisation,^22^ among others. Research into the commercial determinants of health^23 24^ investigates the effect of profit-seeking on health equity, particularly by multinational companies.^25^ Conceptualised as an expansion of the social determinants of health,^26^ the concept lacks political analysis and engagement with political science.^27^

Overall, systematic cooperation between public health and political science disciplines remains rather the exception than the norm.^13 16 28^ A deeper and more productive engagement between political science and public health research could trace and unpack the mechanisms and structures that link global governance with local political dynamics and health outcomes, while also revealing avenues for stimulating change.^13^

Our paper contributes to this broader vision in three ways: First, we operationalise critical political economy theory for the contextualised analysis of political determinants of health and engaging strategically with the politics of policy processes to effect change. We achieve this by introducing a novel theory-based methodological framework. Second, we contribute, through the generic format of the methodological framework, to the ‘structural competency’ of public health experts and to advancing the interdisciplinary cooperation with political scientists. ‘Structural competency’ is the ability to recognise and respond to health outcomes as the result of the political struggle over the course of action that should be adopted to address public health issues.^29^ Finally, we expand the critical political economy scholarship on political determinants of health as we report on the application of the framework to the issue of public health effects of industrial mining in Burkina Faso, Ghana, Mozambique and Tanzania.^30^ Political contestation in mining ranges from the terms of extraction and the fiscal regime to the requirements for environmental and social safeguards, local procurement and employment, and the collection, management, redistribution and reinvestment of revenues.^31^ Public health outcomes are affected by every policy decision, directly (e.g., safeguard policies) or indirectly (e.g., procurement, revenue reinvestment).

The article is structured as follows: We first outline the theoretical foundations of political determinants of health. We then detail the methodological framework before reporting on its implementation in the Health Impact Assessment for Sustainable Development (HIA4SD) research project. We conclude by discussing the implications of our framework for public health research and public health curricula.

## Theory

**Figure 1:**
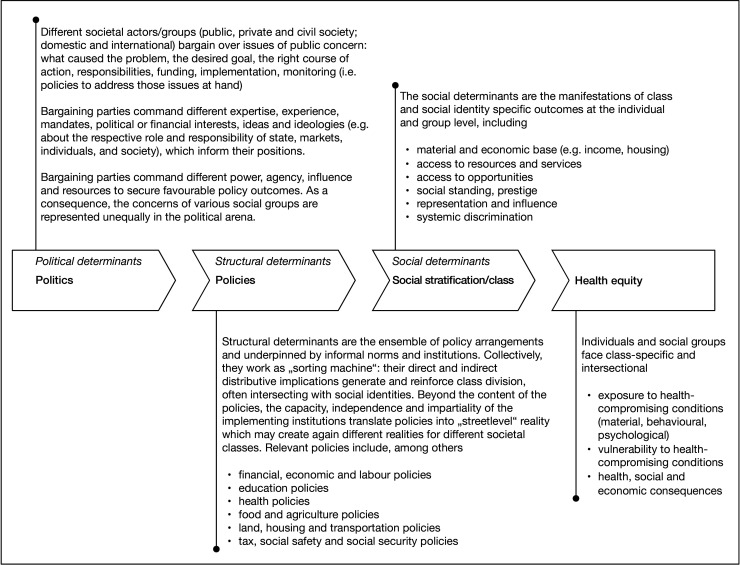
Political determinants of health framework explaining the links between political bargaining processes across policy fields, class formation, and health outcomes. The framework is a conceptual representation to unpack systemic mechanisms and facilitate analysis. In real-world conditions, the different steps are iterative and replete with feedback loops. Source: authors, based on Solar and Irwin^32^.

The World Health Organization (WHO) Commission on Social Determinants of Health provides a helpful starting point for engaging with the political determinants of health. Its 2010 discussion paper^32^ unpacks the social production of disease and the political economy of health, building on Finn Diderichsen’s work on the mechanisms of health inequality.^33^ In this model, health outcomes are mediated in three steps, as shown in figure 1.

First, *political* determinants. Issues of public concern—from providing essential services to security, property rights, the regulation of the economy and the rules of political participation—are continuously (re)negotiated between state and non-state actors. The negotiations are about legitimate knowledge (inquiring about the cause of the issue and the desired status), the right course of action (what needs to be done and when?), responsibility (is it public responsibility at all or rather a private sector or individual responsibility?), funding (who should pay for what?) and implementation (who needs to take action, how to measure progress and how to ensure compliance?). Bargaining parties command different expertise, experience, mandates, interests, ideas and ideologies. Additionally, their agency and resources to secure favourable outcomes vary.

Second, *structural* determinants. Negotiations result in specific policies, regulations and institutions that define the rights, duties and responsibilities of states, organisations and individuals in a particular subject matter area. Collectively and in close interaction with informal cultural and social norms, these policies, institutions and regulations form the structure that defines how societies operate and how individuals and groups interact with one another. These structural determinants function as a ‘sorting machine’. As these structures assign access to material and social resources to some but not others,^34^ they generate or reinforce social stratification, assigning individuals socioeconomic positions within social hierarchies. In the apolitical public health literature, these different positions are described as the social gradient. From a political perspective, this is the (re)production of social class divisions, since the more powerful actors and constituencies who managed to enshrine their preferred course of action into the politically negotiated structures will benefit from greater privileges. Classes often intersect with other characteristics, such as ethnicity, gender, migration and religion, thereby compounding their overall effects on health outcomes. Asking who bears the cost, who gets the opportunities and who faces the risks is a useful entry point to understand whose power and interests structures represent.

Third, *social* determinants. Social stratification leads to different levels of exposure to harmful health conditions, varying susceptibility and differing abilities to protect oneself from health hazards.^32^ Those in ‘lower’ social strata often find themselves in environments with higher exposure to health-damaging conditions such as (environmental) pollution, hazardous jobs and inadequate housing; they frequently have less access to nutritious food, face higher stress levels, have limited access to quality information, lack the ability to afford preventative healthcare and a healthy lifestyle, and often lack the financial means for adequate medical treatment.

Apolitical models, such as the Dahlgren-Whitehead model of health determinants,^35^ focus on visible exposure to health-damaging conditions. Such models hide the political dimension in what they call the ‘general socio-economic, cultural and environmental conditions’, and the lack of progress is reduced to the unhelpful ‘lack of political will’ argument.^36^ Apolitical approaches emphasise individual behaviour and responsibility and call for the ‘empowerment’ of those affected by health inequities.^37^ This places the burden of change on those suffering from structural inequalities, while leaving structures themselves untouched. Consultations are touted as public participation, but are often events where people can express their opinions but cannot effectively participate in decision-making.^38^ Such tokenistic participation serves as a substitute for political change and, in effect, reinforces the status quo, further depoliticising public health.^32^

In contrast, the WHO model addresses the political determinants of health in their own right, including the relevance of non-health policy fields and the importance of the international level. The global governance architecture increasingly influences the socio-political contexts of countries, for example, through the governance of transnational corporations,^39 40^ trade and investment agreements,^41^ donor policies,^42^ multilateral institutions such as the World Bank, the International Finance Corporation^43^ or the International Monetary Fund (IMF),^44^ impacting health determinants, multiplying competing policy priorities, interests and power dynamics.

In the case of public health in industrial mining, the structural determinants include the outcomes from bargaining processes over the terms of extraction. At the national level, the relative priority of promoting extraction to advance the national economy, the social and environmental cost deemed acceptable and the priorities into which revenues are invested. At the local level, the representation and leverage of different social, political and economic actors; the modus of engaging with affected populations during planning, operation and mine closure; the measures deemed appropriate to manage externalities and the relevance assigned to industry self-regulation versus state control, among others. At the global level, the policies of actors with leverage over a country’s policies, such as the IMF imposing austerity measures in return for macroeconomic support or the World Bank pushing the liberalisation of the economy, including the extractive sector. Finally, mining companies negotiate favourable access to resource deposits and promote industry-level self-regulation.

## Methodology

**Figure 2:**
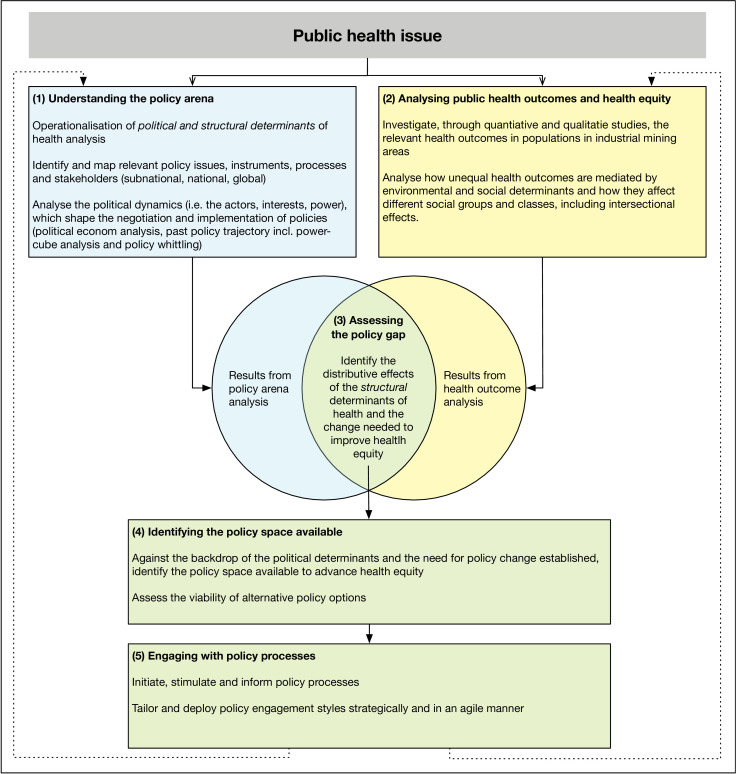
Conceptual overview over the five-step process methodology for comprehensively engaging with the political determinants of health. Steps 1-3 are analytic and deconstruct the politics of health policies; steps 4-5 are strategic, informing policy engagement.

We devise a five-step process for comprehensively engaging with the political determinants of health, building on well-established methods in political and public policy analysis (figure 2). The first three steps are analytical, uncovering the political economy at play. They examine how the ‘sorting machine’, that is, the structural determinants (step 1), lead to class-specific health outcomes, often intersecting with other social identities (step 2), and which elements and arrangements in the structural configuration hinder health equity (step 3). The second part is strategic. Using the knowledge gained during the first part, it identifies effective policy options (step 4) that, considering the political economy at play, have a chance of being adopted (step 5). For detailed information, the methods used are referenced. There has been no patient and public involvement.

### Step 1: exploring policy arena

The policy arena concept operationalises the analysis of the political and structural determinants of health. The policy arena is where bargaining happens and structural determinants are negotiated. As politics always galvanises around specific policy issues, policies serve as entry points to analyse politics. The policy arena^45^ is the space where actors with different interests, priorities and power struggle over which course of action shall be adopted by a government to address the (public health) issue at hand. Such political processes, which ultimately lead to a new or modified policy, do not occur in a vacuum but rather interact with existing social structures, relevant public policies, pertinent institutions, key stakeholders and prevailing power dynamics.^46^

#### Mapping existing policies, institutions and actors

The ensemble of existing policy arrangements in the domain of interest is analysed to understand the current institutional setup and evolution. A (public) policy is the *ensemble* of decisions and intervention programmes of varying legal quality (e.g., hard law, soft law, incentives) undertaken by appointed state institutions, associations or private actors at the subnational, national or international level to solve a societal problem.^47 48^ The *ensemble* includes (1) the goals to be achieved and the legal basis thereof, (2) indicators to measure goal attainment, (3) the policy ‘addressees’, that is, the actors considered relevant for solving the problem and whose behaviour the policy sets out to change or stabilise, (4) the instruments of intervention and (5) the institutional, procedural and financial details of the administrative implementation. The *ensemble* further includes (6) the designation of the agency(ies) in charge and the chain of accountability, (7) the resources allocated for the policy implementation process and (8) the designation of a responsible enforcement authority.^47^ Mapping policies involves reviewing relevant academic and grey literature, analysing regulatory texts and conducting expert interviews.

Mapping the policy arena further requires identifying the public actors (i.e., national and international institutions), societal actors (e.g., civil society, affected populations) and private sector actors (e.g., companies, industry associations) that are involved, interested in or affected by the issue at hand. To get a nuanced understanding of the actors in the policy arena, the list of stakeholders identified is supplemented by (1) their views on what causes the problem (causal hypothesis), (2) how it should be solved (intervention hypothesis), and (3) their agency, that is, the material, organisational and financial resources at their disposal, and their respective networks and capability to mobilise consent or dissent.

#### Analysing the politics in policy processes

A threefold heuristic lens is applied to analyse the politics in and politicisation of the policy process, i.e., how actors deploy resources and agency to represent their interests on seemingly technical questions. The first lens examines policy genesis, asking, ‘How did we get here?’. While apolitical analysis seeks to identify gaps vis-à-vis international best practices, we use a historic-materialist approach that deconstructs political contestation throughout the policy process, explaining the rationale behind suboptimal policies and the interests sustaining them. Different actors are active, playing formal and informal roles, including sometimes contradictory roles, at various points along the policy process. A subtle understanding is critical for anticipating and addressing often hidden resistance to policy change. Policy processes begin with *agenda setting*, where actors with an interest in the topic raise awareness that the problem exists and requires urgent attention. In the *design* phase, actors compete over causal and intervention hypotheses. The emerging design reflects the narrative of the dominant players, with varying degrees of integration of opposing views depending on the relative power of the actors. The resulting policy design is *legitimised* through formal adoption as law, decree, ordinance and so forth. In the case of private regimes (e.g,. sustainability policies of industry bodies or multistakeholder initiatives), legitimation takes the form of standards, codes of conduct or certification and labelling systems. The *rollout*, finally, influences outcomes by shaping how policies are interpreted, implemented, monitored and enforced. Methods such as process tracing^49^ and discourse network analysis^50^ among others help identify causal chains and reveal how actors build coalitions.

The power cube framework, the second heuristic lens, analyses how power operates in negotiation and decision-making along the policy process.^51^ *Spaces of power* refers to who controls the negotiation space. *Closed* spaces are controlled by an elite group that defines the issues discussed and controls the decision-making process without broader involvement. *Invited* spaces are still controlled by an elite group but open to selected actors from the policy arena to discuss issues of mutual interest. *Claimed* spaces are opened by less powerful actors, for example, by creating their own fora in a way that can no longer be ignored by powerful actors or by negotiating access to invited or closed spaces. *Levels of power* unpacks how the local, national and global fora of negotiation and decision-making are interlinked. Global levels have become more relevant, but they are also more difficult to access. *Forms of power* includes *visible* power assigned through roles and institutions, *hidden* power as the actual control over the process and decision-making and *invisible* power, exercised through discursive hegemony, which is the ability to dominate the way of talking about or understanding an issue so that it influences how individuals think of the issue, thereby sidelining alternative views.

The third heuristic lens identifies instances of *policy whittling*. Actors typically promise comprehensive solutions once an issue reaches the political agenda. However, bargaining often narrows the scope addressed, shifting responsibilities to private actors or citizens. Legal, economic or political costs may drive decision-makers to adopt weaker measures, reducing policy effectiveness. Similarly, negotiation may exclude key actors if they resist and wield sufficient power. *Whittling down* also occurs during implementation, whether by ignoring facts necessitating action or selectively overlooking policy failures in evaluations.

### Step 2: analysing public health outcomes

Analysing public health outcomes seeks to clarify the effect of existing policies and their implementation on the health of different societal classes and their intersection with different social identities. This requires diverse and complementary data on health determinants and outcomes to identify relevant impacts and investigate potential health equity issues, as exemplified by the study protocol of the HIA4SD research project.^52^ Methodologically, the purposeful integration of quantitative analysis with qualitative investigation provides significant advantages, particularly in gaining a deeper understanding of the public health dynamics and the influence of political determinants of health.^53^ Quantitative analyses capture broad patterns and averages across populations or regions at an aggregate level, potentially masking localised or subgroup disparities. Qualitative investigations offer deeper localised insights that might reveal hidden inequalities, explain how benefits and harms are distributed unevenly across communities and explore intangible or non-biomedical dimensions of health such as perceived injustice, community disempowerment, alienation or longer-term, systemic harms. A careful mixed-methods design will use qualitative findings to interpret and refine quantitative results, for example, by identifying overlooked indicators or guiding disaggregated quantitative analysis at finer spatial or socio-demographic scales to reveal nuances that aggregate statistics may obscure. Qualitative analysis, in turn, can benefit from quantitative insights, for example, to refine research questions, inform sample selection, explore causal mechanisms and contextual factors behind quantitative findings or validate or challenge findings by capturing lived experiences that put statistical patterns into a broader perspective.

### Step 3: assessing the policy gap

The joint assessment of the results from the first two steps uncovers the distributive effects of existing structural determinants, their impact on individuals along societal class divisions and social identities, often creating intersectional compound effects, and highlighting the need for policy change. Key guiding questions include: What and how severe are the identified public health issues, and for whom? What positive outcomes should be preserved or strengthened? What are the pathways through which the ensemble of policies, their modus of implementation and further incentives at work influence health outcomes, directly and indirectly? The evidence gathered helps identify changes in and the evolution of the structural determinants and policies required to improve public health outcomes. Yet, since policies are always the result of a political process involving deliberation, interest representation and power struggles, the question emerges: ‘How can we get there?’.

### Step 4: identifying the policy space to modify structural determinants

We refer to the ‘policy space’ as the domain where actors with political influence in the policy arena share sufficient common ground to work towards modifying the structural determinants so that the ‘sorting machine’ leads to more equal outcomes. To identify this policy space, we analyse the existing academic, practitioner and political discourse to identify the range of policy options that can contribute to improving health outcomes. This range of theoretically possible policy options provides the basis for eliciting the perspectives, preferences and priorities of actors across the political economy dynamic, allowing for the detection of overlap and contradiction. One research technique is Q-methodology,^54^ other methods include force field analysis, discourse network analysis or preference surveys. Insights from the political economy analysis (step 1) help to put the results into perspective.

### Step 5: engaging with policy processes

Since policymaking involves strategic negotiation about what causes a problem and how to address it, the way the negotiation process unfolds matters for the outcome. Political processes can be nurtured and advanced by skillfully combining the six engagement styles^48^ presented below in response to how the process evolves.

Academics are familiar with the *research-and-analysis* engagement style: conducting studies that contribute scientific rigour, validity and reliability to the issue at hand. While this allows for political deliberations to be grounded in high-quality evidence, it does not guarantee evidence-based policy outcomes. The *design-and-recommend* style derives actionable policy options from available evidence. The *strategic-advising* engagement style navigates complex political landscapes and proactively tailors communication, lobbying and advocacy to advance a particular position in a specific policy arena. The *mediation* engagement style fosters convergence by facilitating shared understanding, negotiation and agreement among diverse stakeholders. The *democratisation* style is deployed to strengthen the legitimacy of a policy process by ensuring openness, transparency and equal representation in the deliberation. Lastly, the *clarifying-values-and-arguments* style aims at improving the depth and quality of debates, ensuring that discussions remain logically sound, well-structured and inclusive of diverse perspectives. Selecting the engagement style—or combination of styles—that best responds to the unfolding deliberation dynamics is the art, not science, of engaging with policy processes.

## Results

The following section draws on a large body of previously published work in the context of the interdisciplinary HIA4SD research project into public health effects of industrial mining,^30^ conducted by epidemiologists and political scientists over a 6-year period in Burkina Faso, Ghana, Mozambique and Tanzania. We summarise the key insights and results to illustrate how the overarching methodology guided the engagement with political determinants of health. Details on the specific methods, data collection and analysis are reported in the referenced articles.

### The policy arena

Foreign direct investment in industrial mining is actively promoted by multilateral development banks, including the World Bank, as a strategy to expand fiscal space, stimulate economic growth, create employment and generate hard currency. Investment treaties^55^ and investor-state dispute settlement mechanisms^56^ serve to attract and protect investments. Mining companies have also used these regulations to sue host states for protecting the environment or addressing social concerns.^57^,^59^ The primacy of promoting investment weakens the country’s negotiating position and vigorous insistence on environmental and social safeguards.

The management of public health in industrial mining is organised in layers. At the national level, environmental impact assessments (EIA) are the key instrument for identifying, mitigating and managing externalities. International EIA blueprints^60^ have been introduced across Africa since the 1990s as part of liberalisation reforms to attract foreign investment.^61^ While social impacts are frequently mentioned in EIA regulations, public health is not part of mandatory EIAs across Africa.^62^ Country institutions tasked with EIA implementation and monitoring, typically semi-autonomous entities or units in the Ministry of Environment, while decision-making power regarding mining licenses is vested in the highest echelon of the government. Our political economy analysis, based on expert interviews and review of the literature, reveals that the technical bodies lack capacity and clout.^63^,^65^ Insufficient resources for properly functioning environmental units are found to be largely intentional, and their recommendations are frequently overruled.^66^,^68^ Community consultations resemble tokenism, offering the appearance of inclusion without meaningful influence.^69^ Insufficient environmental and social protection stems largely from flawed legal implementation, raising concerns that new regulations may face the same fate.

The national level interacts with global regimes. The International Finance Corporation requires loanees and investees to conduct health impact assessments (HIA).^70^ Private banks subscribing to the Equator Principles likewise mandate it in project finance, but their leverage weakened after key signatories withdrew in 2022.^71^ The Extractive Industry Transparency Initiative (EITI) requires participating countries to publish EIAs and monitoring reports, encourages the disclosure of underlying environmental and social monitoring data, and sanctions imposed. Although all HIA4SD study countries are EITI members, none disclose data on monitoring or sanctions. Finally, the mining industry’s sustainability body, ICMM, encourages HIA and provides practice guidance. Yet, none were publicly available in our study countries.

**Figure 3:**
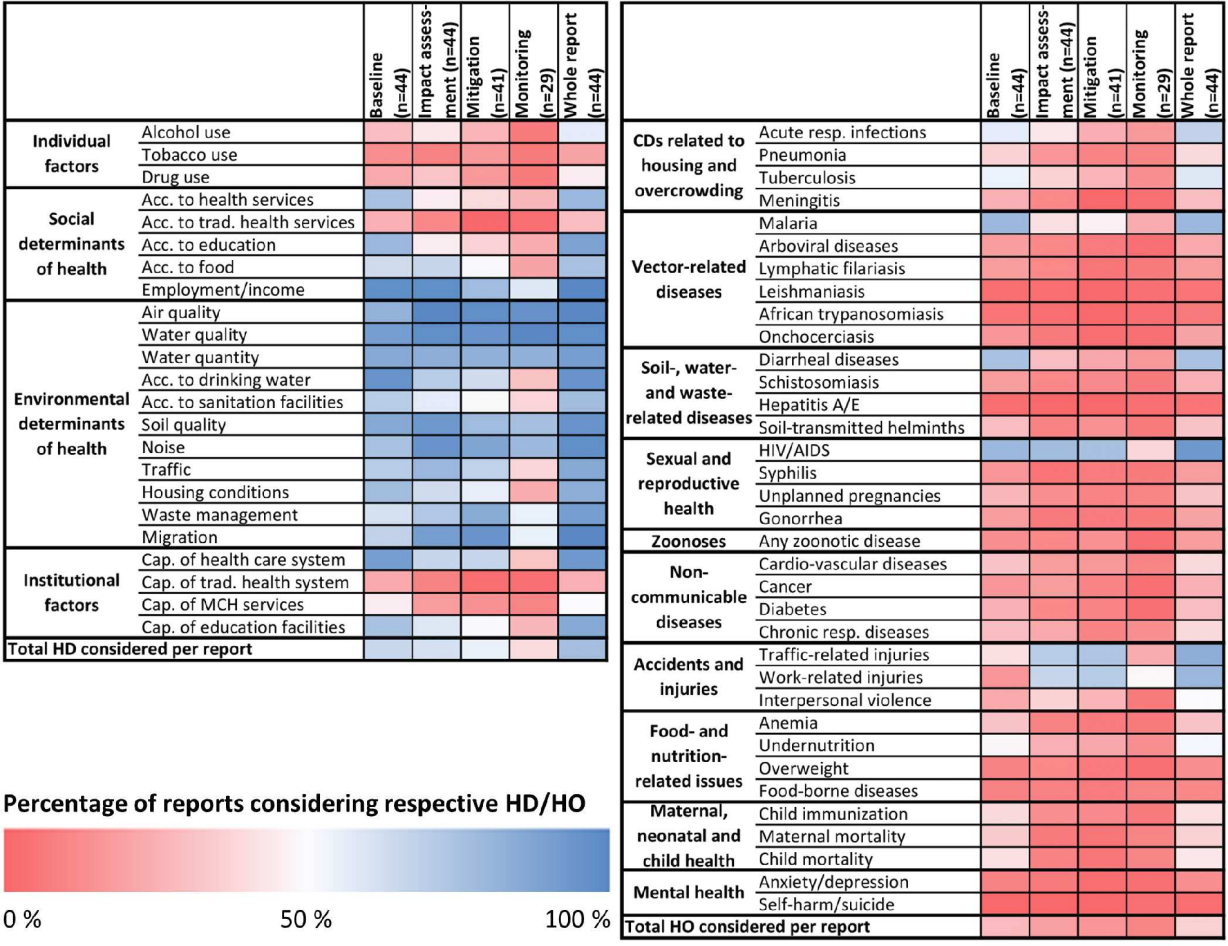
Inclusion of health determinants (HD; left panel) and health outcomes (HO; right panel) in impact assessment reports. Colors represent the percentage of reports or report sections considering the specific health aspect. Red shading indicates percentages below 50%, blue shadings above 50%. Acc., access; Cap., capacity; CD, communicable disease; MCH, maternal and child health; resp., respiratory; trad., traditional. Source: Dietler et al.^72^.

Voluntary private-governance regimes also show limited efficacy. Of the 569 mining projects across Africa, we were able to access only 44 EIAs.^72^ Of those, most consider environmental determinants of health; social and institutional factors, such as the accessibility and capacity of health services or maternal and child health services, were rarely included (figure 3).^72^ This also relates to the breadth and rigour of the evidence used. In most cases, mining companies use secondary data on health outcomes from routine health monitoring systems without systematically determining the need for primary data, as recommended by HIA international best practice.^73 74^ Consequently, evidence on health determinants is notably absent in the sections defining mitigation measures, the management plan and the monitoring strategy.^72^

We then applied Qualitative Comparative Analysis (QCA) to a subsample of 18 EIA reports from the four study countries to identify what drives self-regulation.^75^ Our results suggest that risk mitigation predominates: conflict risk and an open civic space are both linked to a greater inclusion of public health concerns in EIAs. This transactional approach is mirrored in corporate spending patterns. Our 8-year panel of 292 mining projects in 17 African countries suggests that mining companies with recent exposure to local conflict are more likely to fund community initiatives. Social spending modestly eases tensions, but the effect dissipates after a few years.^76^

### Public health impacts

**Figure 4:**
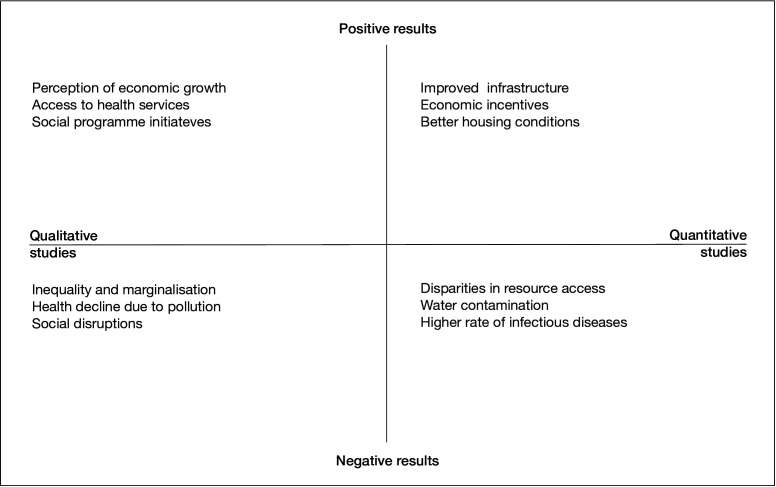
Schematic overview of the positive and negative results observed in the quantitative and qualitative studies conducted in the frame of the HIA4SD research project. Source: authors.

Our mixed-methods analysis^52^ of public health outcomes reveals positive and negative effects. Overall, the quantitative studies identified significant correlations between mining activities, social and environmental health determinants and associated health outcomes (this is further elaborated on in the next paragraph). The qualitative studies added depth, capturing personal and social dimensions, including the critical dimension of perceived impacts,^77^ highlighting the need to combine both to fully understand these effects.^78^ A cross-cutting finding is that the health benefits of mining projects are unevenly distributed, primarily benefiting wealthier households (Figure 4).

For example, the analysis of data from over 180 000 households within a 100 km radius around mining sites in 27 African countries found that improved access to safe water and sanitation infrastructures after mine opening was, on average, considerably larger in households near mining sites than in comparison areas located further away.^79^ Similarly, household wealth, a central determinant of health, increased more rapidly in communities directly impacted by a large-scale copper mine in rural Zambia than in comparison communities.^80^ Positive effects on health outcomes observed in the quantitative studies were, for instance, that the opening of mining projects reduced the mortality risk among neonates^81^ and the prevalence of stunting and underweight in children under 5 years of age.^79^ Adverse health outcomes found in quantitative studies include an almost twofold increase in HIV infection following mine openings, associated with a tendency towards lower HIV knowledge and increased odds of risky sexual behaviour.^82^ Evidence from a prospective mortality surveillance study in Tanzania found that mining workers had over twice the mortality risk relative to non-miners living in the same communities, with death due to road traffic accidents being the primary cause.^83^

Qualitative studies revealed a broader range of impacts. While local communities acknowledge the social and economic benefits of interventions by the mining companies, such as new or improved water sources and healthcare facilities, the perceived negative impacts (e.g., environmental pollution and social disruption) dominate,^84^ with women disproportionately affected.^85^ Complementary to gendered impacts, inequities related to individual characteristics (e.g., migrants vs local populations),^86^ intermediate factors acting at the community level (e.g., environmental pollution and land loss) and structural conditions (e.g., access to jobs and services) emerged as central topics.^85^

The results show that mining projects systematically have unequal impacts on different population groups. While the wealthier households benefit from positive health impacts, poorer households, women and migrants are most exposed to adverse effects on health determinants and health outcomes. In addition to the unequal effects observed, stakeholders in mining areas have contradictory perceptions of mining-related health effects. For instance, while mining officials in Burkina Faso reported mainly positive effects, healthcare providers and community leaders primarily reported adverse health impacts on the health of affected communities, specific health determinants and healthcare service provision.^87^

### Policy gap

The combined analysis of the health outcome patterns with the structural determinants reveals the following situation: While the regulatory ensemble stipulates the systematic consideration of health impacts that considers the situation and needs of all population groups irrespective of class, gender and other group characteristics,^72 73^ the absence or voluntary characteristics of health-specific requirements allow for an eclectic consideration, guided by a functionalist logic that prioritises the operational imperatives of extractive companies. In parallel, the governments’ prioritisation of foreign investment is coupled with defunding the administration’s capacity to monitor and implement existing EIA regulations. Hence, the existing regulatory structure fails to deliver on the adopted public health management goals, at the expense of those who are least represented at the political level.

### Policy space

Is there sufficient common ground among political contenders to advance change? Our Q-studies^88^,^90^ find that, across all four countries, public actors at the national and local levels, the private sector and civil society cluster around two policy perspectives. First, all stakeholders in all countries reject policy options that maintain the status quo. Such fundamental agreement is conducive to initiating a policy process. Second, the appetite for fundamental legal reform is limited. Only the two perspectives in Ghana and one in Burkina Faso prioritise comprehensive legal reform, either by introducing an HIA law or by including public health in EIA legislation. All other perspectives do not prioritise legal reform, suggesting that they do not attribute the current underperformance to missing legal provisions. This is relevant because donors often suggest legal reforms to address issues identified.

Third, all countries prioritise implementation-focused measures. Including public health in the companies’ management plan, collecting baseline data for effective monitoring and the possibility of imposing effective fines consistently rank among the highest priorities. The emphasis on monitoring and compliance reflects the experience that EIA performance does not suffer from missing regulations but from an implementation deficit. Fourth, all perspectives in all countries explicitly rule out the idea that the private sector self-regulates. Private governance is supported by donors, international organisations and the industry, but is not recognised by host country actors as a legitimate and credible custodian of public health due to conflicts of interest. However, both perspectives in Tanzania and one in Mozambique call for more funding from mining companies for the local health system. This view is particularly emphasised by study participants from subnational levels, where the mining companies’ capacity and resources are part of the daily experience.

Finally, important opportunities and tensions emerge, too. Private sector representatives signal varying degrees of openness to accepting responsibilities for protecting public health. Fault lines emerge within the public sector. The first falls between those tasked to promote foreign direct investment in mining and those concerned with mitigating negative externalities. Leveraging the previously observed support from the industry could help overcome this divide. Second, friction is visible between the Ministries of Environment, responsible for environmental monitoring, and the Ministries of Health, as they might compete for funds, staff and influence. Finally, tensions arise between the central and local levels over the competencies that local-level health system actors should be entrusted with for monitoring and enforcement.

### Policy engagement

In each country, the local HIA4SD research partner initiated and nurtured a country-specific policy process. Following the basic sequencing of policy processes, the project held regular country-level stakeholder meetings from the outset to raise awareness and involve local actors. A second focus was on technical workshops with ministries, academia and the private sector to familiarise them with HIA. Third, the project convened stakeholders to broaden support, clarify arguments and mediate between differing positions. For example, in Tanzania, the revision of the Public Health Act provided an opportunity to introduce HIA concepts by sharing research insights, designing and recommending guidelines and mediating between stakeholders. In Mozambique, the continuous exchange and sharing of results opened the opportunity to discuss HIA concepts and their inclusion in the EIA guidelines revision. In Ghana, the early involvement of the Environmental Protection Agency and the Ghana Health Service proved effective in agenda setting and convening interested parties and decision-makers in various configurations to clarify possible pathways and efforts required towards mandatory HIA. In all countries, successfully navigating policy dynamics involves accurately ‘reading’ the evolving dynamics, seizing opportunities and making agile decisions about when and how to employ different engagement styles were detrimental to advance the process. After all, policy engagement is as much an art as a science.

## Discussion and conclusion

The 6-year, multicountry HIA4SD research project is one of the few projects that systematically integrated public health research with political analysis and policy engagement. While the framework worked well as conceptual and methodological guidance, more effort was needed to ensure the interdisciplinary cooperation in the country-level analysis of the policy arena (step 1) and the policy engagement (steps 4 and 5); a combination of introduction into basic concepts, methodological training, joint investigations, regular check-ins and coaching from seasoned local health policy actors helped to build capacity incrementally. In retrospect, this applied learning proved effective, as one epidemiologist reflected on his journey: “I have developed as a facilitator of the policy process. Knowing ‘how the machine works’ makes me listen better and understand what is happening. While I resisted the task first, I now enjoy the policy engagement”.

The findings are relevant beyond this project. First, the proposed framework to engage with the political determinants of health can advance the policy impact of public health research where equity is a concern^91^ and social determinants play a significant role, from access to healthcare^92^ to non-communicable diseases,^93^ infectious diseases^94^ to nutrition,^95^ child^96^ and maternal health,^97^ to mention just a few. The proposed framework also benefits implementation research,^98^ as most implementation studies in low- and middle-income countries have been conducted under conditions where the researchers have considerable influence over implementation rather than in ‘real world’ conditions,^99^ de facto excluding political determinants and seriously limiting the applicability of findings.^100^

Second, strategically enhancing the policy relevance of public health research requires institutionalising substantive collaboration between political scientists and public health experts. In the best case, this ambition is met by an increasing demand from funders, development agencies and government actors seeking to understand political dynamics and find answers to promote policy-making to realise health equity.

Finally, future public health scholars and practitioners need stronger ‘structural competence’, i.e., training in understanding and engaging with policy processes. Knowing ‘how the machine works’ is critical to equipping public health experts with the basics to translate public health research findings into effective policy engagement. Hence, revising curricula for public health expert education to include training not only in sociological analysis but in politics and public policy is a prerequisite to increasing policy relevance.

## Data Availability

All data relevant to the study are included in the article or uploaded as supplementary information.
